# Insights into the mechanism of intestinal flora imbalance and immune disorder in co-morbidity of pneumonia and diarrhea in children

**DOI:** 10.3389/fped.2026.1836762

**Published:** 2026-06-12

**Authors:** Min Li, Zaifang Sun, Tiezhu Jia, Mingli Ma

**Affiliations:** 1 Department of Pediatrics, Jinan Third People’s Hospital, Jinan, China; 2 Clinical Laboratory Center, Jinan Third People’s Hospital, Jinan, China

**Keywords:** clinical laboratory testing, comorbidity, diarrhea, gut microbiota, gut-lung axis, immune disorders, pediatric pneumonia

## Abstract

Pneumonia and diarrhea are the two leading causes of death in children under five years of age, and these two conditions often present as a comorbidity, where the same child experiences respiratory and digestive system infection symptoms simultaneously or sequentially. Clinical data indicate that the incidence of secondary diarrhea in children hospitalized with pneumonia is high, significantly prolonging hospital stays and affecting prognosis. In recent years, the proposal of the gut-lung axis theory has provided a novel perspective for understanding this comorbidity phenomenon. The gut-lung axis refers to the bidirectional regulatory pathway between the gut microbiota and the pulmonary immune system, with the lungs and intestines sharing embryonic origin and a common mucosal immune system. This review systematically reviews the characteristics of gut microbiota dysbiosis and the mechanisms of immune disorders in the context of pediatric pneumonia-diarrhea comorbidity. Clinical studies have shown that children with comorbidity exhibit significant gut microbiota dysbiosis, characterized by a reduction in beneficial bacteria such as *Bifidobacterium*, an increase in opportunistic pathogens such as *Escherichia coli*, and decreased microbial diversity. Gut microbiota dysbiosis leads to immune disorders through multiple mechanisms, including reduced short-chain fatty acids, skewed immune cell differentiation, and dysregulated inflammatory factor networks, resulting in Th1/Th2 imbalance, decreased regulatory T cell function, and exacerbated systemic inflammatory responses. Supplementation with microecological preparations such as *Saccharomyces boulardii* has been shown to significantly shorten hospital stays, diarrhea duration, and fever resolution time, while improving peripheral blood immunoglobulin levels and T-cell subsets, providing evidence-based support for clinical intervention. This review also systematically reviews clinical laboratory indicators associated with comorbidity, including inflammatory markers, immune status indicators, intestinal barrier function markers, and microbiota detection methods, which have important application value in early identification, disease assessment, and treatment monitoring of comorbidity. Future research should further employ metagenomic approaches combined with longitudinal follow-up designs to elucidate the roles of specific bacterial species/strains in gut-lung axis regulation, providing new strategies for precision prevention and treatment of pediatric pneumonia-diarrhea comorbidity.

## 1 Introduction

Pneumonia and diarrhea are two common diseases that threaten the health of children worldwide. According to the World Health Organization, they are the two leading causes of death in children under five years of age, accounting for nearly one-third of all deaths in this age group (1). Clinically, these two diseases often present as a comorbidity, where the same child experiences respiratory and digestive system infection symptoms simultaneously or sequentially (2). This not only prolongs hospital stays and increases medical costs but also affects the child's nutritional status and immune function recovery. Globally, the comorbidity of pneumonia and diarrhea is not uncommon in children. A community-based cross-sectional study in Ethiopia showed that the prevalence of comorbid diarrhea and respiratory infection symptoms in children under five years old was 17.2% (3). The incidence of comorbidity is even higher among children hospitalized with pneumonia in China (4). A study involving 1,352 children with bronchopneumonia found that the rate of secondary diarrhea was as high as 39.4%. Another retrospective cohort study on children with severe pneumonia reported a 13.7% incidence of antibiotic-associated diarrhea, with a median time of 5 days from antibiotic administration to the onset of diarrhea (4). These data suggest that the comorbidity of pneumonia and diarrhea is a widespread clinical issue, both in community and hospital settings.

In children hospitalized with pneumonia, the comorbidity state presents more complex clinical characteristics. Studies have shown that children with *Mycoplasma pneumoniae* pneumonia complicated by diarrhea have a longer disease course, prolonged fever, and extended duration of antibiotic use. Additionally, their levels of inflammatory factors such as interleukin (IL)-4, IL-6, and TNF-α are significantly higher than those in children with pneumonia alone, indicating a more severe condition (5). Multivariate analysis has revealed that the duration of hospitalization, disease severity, combined use of multiple antibiotics, invasive procedures, duration of antibiotic use, age, and mechanical ventilation are independent risk factors for secondary diarrhea in pneumonia (6, 7). Conversely, older age, handwashing before meals, higher neutrophil ratio and hemoglobin levels, and the use of microecological preparations serve as protective factors. Prophylactic use of probiotics has been confirmed to significantly reduce the risk of comorbidity, providing an important direction for clinical intervention.

For a long time, the medical community has regarded the respiratory and digestive systems as functionally independent organ systems, making it difficult to explain the pathogenesis of pneumonia-diarrhea comorbidity. However, clinical observations have revealed that respiratory syncytial virus (RSV) infection is often accompanied by gastrointestinal symptoms, and children with rotavirus enteritis frequently present with respiratory manifestations, suggesting an unrecognized pathophysiological connection between them (8, 9). In recent years, the introduction of the gut-lung axis concept has provided a novel theoretical framework for understanding this relationship (2). The gut-lung axis refers to the bidirectional regulatory pathway between the gut microbiota and the pulmonary immune system. The lungs and intestines share embryonic origin and a common mucosal immune system. The gut microbiota not only maintains intestinal homeostasis through local immune regulation but also remotely influences pulmonary immune function via metabolites such as short-chain fatty acids (SCFAs), and the migration of immune cells. Recent clinical studies have also provided direct evidence for this theory. It has been found that children with severe pneumonia exhibit significant gut microbiota dysbiosis, characterized by markedly lower fecal counts of *Bifidobacterium* and a reduced *Bifidobacterium*-to-*Escherichia coli* ratio compared to healthy controls, along with significantly increased counts of *Escherichia col*i (10). The core pathological changes, key molecular mediators, and corresponding clinical laboratory indicators are summarized in Table 1. The B/E ratio was negatively correlated with peripheral blood inflammatory factors, such as TNF-α and TLR4, and positively correlated with antioxidant factors, directly confirming the association between gut microbiota dysbiosis and systemic inflammatory response. Intervention studies have further validated this causal relationship (11, 12).

**Table 1:** Core pathological changes, key molecular mediators, and corresponding clinical laboratory indicators in pediatric pneumonia-diarrhea comorbidity.

Increased opportunistic pathogens	Core pathological changes	Key molecules	Clinical laboratory indicators
Gut Microbiota Dysbiosis	Decreased microbial diversity	a-diversity indices	16S rRNA sequencing, metagenomic sequencing
	Reduction in beneficial commensal bacteria	*Bifidobacterium*, *Lactobacillus*, *Faecalibacterium*	*Bifidobacterium* count, B/E ratio
	Increased opportunistic pathogens	*Clostridium difficile*, *Klebsiella pneumoniae*, *Enterococcus*	Pathogen culture, qPCR. toxin detection
Abnormal SCFA Metabolism	Reduction in SCFA-producing bacteria	*Faecalibacterium prausnitzii*, *Eubacterium rectale*, *Bifidobacterium adolescentis*	Fecal SCFA detection (acetate, propionate, butyrate)
	Decreased SCFA levels	Butyrate, propionate, acetate	Serum/fecal SCFA quantification
Th1/Th2 Immune Imbalance	Th1/Th2 deviation	IL-2, IFN-γ, IL-4, IL-5, IL-13	Serum cytokine profiling
Dysregulation of Inflammatory Cytokine Network	Elevated pro-inflammatory cytokines	TNF-a, IL-6, IL-1B	CRP, SAA, IL-6, PCT
	Mechanical barrier damage	Tight junction proteins (occludin, ZO-1)	Serum D-lactate, diamine oxidase, endotoxin
Dysregulation of Inflammatory Cytokine Network	Immune barrier damage	Secretory IgA	Fecal SIgA
	Chemical barrier damage	Mucin	Fecal mucin

A randomized controlled trial involving children with pneumonia complicated by secondary diarrhea showed that, compared with the use of montmorillonite powder alone, adjuvant therapy with *Saccharomyces* significantly shortened the duration of hospitalization, diarrhea, and fever (13). Following treatment, the adjuvant therapy group exhibited significantly higher levels of peripheral blood immunoglobulins, T cell subsets, and counts of *Enterococcus* and *Lactobacillus* compared to the control group, confirming that regulating the gut microbiota can improve immune function and promote clinical recovery. Based on the above background, this review aims to systematically summarize the mechanisms of gut microbiota dysbiosis and immune disorders in the context of pediatric pneumonia-diarrhea comorbidity. It will focus on elucidating the central role of the gut-lung axis in the occurrence of comorbidity, analyze the key pathways through which microbiota dysbiosis leads to immune dysregulation, and discuss the application value of clinical laboratory indicators in the diagnosis, severity assessment, and treatment monitoring of this comorbidity, thereby providing a scientific basis for early identification and precision intervention in clinical practice. The clinical application value of laboratory indicators in pediatric pneumonia-diarrhea comorbidity is summarized in Table 2.

**Table 2:** Clinical application value of laboratory indicators in the diagnosis, severity assessment, and treatment monitoring of pediatric pneumonia-diarrhea comorbidity.

Indicator category	Specific indicators	Detection methods	Clinical application value
Inflammatory Markers	CRP, SAA, IL-6, PCT, WBC	Immunoturbidimetry, ELISA, Chemiluminescence	Early infection recognition; Bacterial/viral differentiation; Dynamic disease monitoring
Nutritional and Immune Status	Prealbumin, Zinc, Ferritin	Biochemical assays, Atomic absorption spectroscopy	Identification of high-risk children; Guidance for nutritional intervention; Treatment efficacy evaluation
	Delayed-type hypersensitivity	Multi-antigen skin testing	Predicts gastrointestinal and respiratory infection risk
Intestinal Barrier Function	D-lactate	ELISA	Early identification of increased intestinal permeability
	Diamine oxidase	ELISA	Reflects degree of intestinal mucosal cell damage
	Endotoxin	Turbidimetric assay	Assesses bacterial translocation and systemic inflammation risk
Gut Microbiota Quantification	*Bifidobacterium* count	qPCR, Culture	Assesses probiotic levels
	*Escherichia coli* count	qPCR, Culture	Assesses opportunistic pathogen burden
	B/E ratio	Calculation (log *Bifidobacterium*/log *E. coli*)	Core indicator of colonization resistance; B/E < 1 indicates dysbiosis
Intestinal Immune Barrier	Secretory IgA	ELISA	Assesses intestinal mucosal immune function
	Mucin	ELISA	Assesses intestinal chemical barrier function
High-throughput Microbiota Analysis	16S rRNA sequencing	High-throughput sequencing	Comprehensive microbiota structure analysis; Identification of specific genera changes; Long-term prognosis prediction
	Metagenomic sequencing	High-throughput sequencing	Functional gene analysis; Resistance gene detection; Personalized microbiota intervention guidance
Pathogen Detection	*Enteric virus* detection	PCR, Antigen detection	Identifies etiological basis of comorbidity
	*Clostridium difficile* toxin	ELISA, PCR	Diagnoses antibiotic-associated diarrhea

## 2 The gut-lung axis

### 2.1 The concept and biological basis of the gut-lung axis

The gut-lung axis refers to the bidirectional regulatory network between the intestinal microecosystem and the pulmonary immune system. The gut-lung axis refers to the bidirectional regulatory network between the gut microbiota and the pulmonary immune system. It is crucial to note that the respiratory tract itself harbors a unique microbial community, the lung microbiota, which also plays an active role in this axis. However, given that the biomass of the lung microbiota is orders of magnitude lower than that of the gut and is largely derived from microbial translocation from the oral cavity and the gut, the gut microbiota is considered a primary driver of systemic immune modulation. Therefore, this review will focus on the established mechanisms by which the gut microbiota and its metabolites remotely regulate pulmonary immune homeostasis, while acknowledging the emerging role of the lung microbiota. Its theoretical framework is constructed upon three core dimensions, embryonic developmental homology, shared mucosal immune system, and microbial metabolite-mediated signal transduction. The introduction of this concept provides an integrated theoretical framework for elucidating the comorbidity mechanisms of respiratory and digestive system diseases. Embryonic homology constitutes the anatomical foundation of the gut-lung axis (14). Both the lungs and the large intestine originate from the primitive foregut, sharing a common germ layer origin during embryonic development. This developmental homology determines their intrinsic connection in tissue structure and physiological function, resulting in a high degree of pathological correlation between the respiratory and digestive systems under disease conditions.

The common mucosal immune system serves as the functional core of the gut-lung axis. The intestinal tract and respiratory tract both belong to the mucosal immune system and share immune cell homing and recirculation mechanisms (15). Activated lymphocytes from the intestinal mucosa, particularly T cells expressing α4β7 integrin, can enter the circulation via the thoracic duct and directionally home to the lamina propria of the respiratory mucosa, performing immune surveillance and effector functions (16, 17). This common mucosal immune system theory explains how alterations in local intestinal immune status can remotely regulate respiratory immune function. The gut microbiota, as the core effector of the gut-lung axis, exerts its biological effects through three primary mechanisms.

First, the gut microbiota produces metabolites such as SCFAs, which enter the circulation and regulate pulmonary immune function (18). Secondly, the gut microbiota regulates immune cell differentiation regulation. The gut microbiota regulates the differentiation of naive T cells into distinct subsets by providing antigenic stimulation and metabolic signals. Specific commensal bacteria such as segmented filamentous bacteria can induce Th17 cell differentiation, while *Clostridium* clusters IV and XIV promote regulatory T cell generation (19, 20). These cells migrate to the respiratory tract through homing mechanisms and participate in the maintenance of local immune homeostasis. Thirdly, the gut microbiota modulates mucosal barrier function. The gut microbiota maintains intestinal epithelial barrier integrity by regulating the expression of tight junction proteins involving occludin, claudin, and ZO-1 and mucus secretion (21, 22). Barrier dysfunction can lead to the entry of bacterial products such as lipopolysaccharide into the circulation, activating systemic inflammatory responses and indirectly affecting pulmonary immune function.

### 2.2 The regulatory role of gut microbiota in respiratory immunity

As the core effector of the gut-lung axis, the gut microbiota remotely regulates respiratory immune function through multiple mechanisms, a regulatory role that has been substantiated by extensive basic and clinical research.

At the animal experimental level, studies have provided direct evidence for the immunomodulatory function of the gut microbiota. Probiotic intervention studies have demonstrated that oral administration of *Lactobacillus delbrueckii* UFV-H2b20 significantly enhances resistance to pulmonary *Aspergillus fumigatus* infection in mice (23). The underlying mechanism involves the induction of tolerogenic dendritic cells, IL-10-positive macrophages, and FoxP3-positive regulatory T cells in mesenteric lymph nodes, accompanied by increased fecal IgA levels (24, 25). Following infection, the intervention group exhibited significantly improved survival rates, markedly reduced lung fungal burden, decreased pulmonary vascular permeability, and increased numbers of FoxP3+ regulatory T cells within the lungs, along with elevated levels of TGF-β and IL-10, while pro-inflammatory factors such as IL-1β, IL-17A, and CXCL1 were reduced. Fecal microbiota transplantation experiments have further confirmed the protective role of gut microbiota, transferring gut microbiota from H7N9 influenza survivors to recipient mice significantly enhanced the latter's resistance to influenza virus infection (26, 27). SCFAs, as key metabolic mediators, have been extensively validated for their immunomodulatory effects. Butyrate promotes regulatory T cell differentiation and inhibits Th2-type inflammatory responses, thereby alleviating respiratory inflammatory injury (28, 29).

At the clinical research level, associations between gut microbiota composition and susceptibility to respiratory infections have been elucidated. A prospective study of patients with community-acquired pneumonia revealed that, compared to healthy controls, patients with pneumonia exhibited significantl**y re**duced gut microbiota α-diversity and distinct differences in β-diversity (29). Short-chain fatty acid-producing genera, such as *Blautia* and *Faecalibacterium*, were markedly decreased, while opportunistic pathogens including *Gemmiger* and *Clostridium* were increased. Children with recurrent respiratory tract infections similarly showed reduced gut microbiota diversity, with increased *Enterococcus* and decreased *Eubacterium*, *Faecalibacterium*, and *Bifidobacterium*. Analysis of gut microbiota in children with respiratory syncytial virus infection revealed increased proportions of *Muribaculaceae*, *Clostridium*, and *Lactobacillus*, with severe cases exhibiting higher relative abundance of *Muribaculaceae* compared to moderate cases. Studies in children with asthma have revealed that early-life gut microbiota dysbiosis constitutes a critical risk factor for asthma development.

Lower gut microbial diversity at one month of age correlates with higher asthma risk during school age, with increased abundance of *Streptococcus*, *Bacteroides*, and *Clostridium*, along with decreased *Bifidobacterium* and *Faecalibacterium*, being significantly associated with asthma risk. Clinical intervention studies have further validated the value of modulating gut microbiota in preventing and treating respiratory infections. Oral probiotic administration has been shown to reduce the risk of respiratory failure in patients with COVID-19 (30). Children with recurrent respiratory tract infections supplemented with probiotics exhibited significantly decreased infection frequency. In a COPD animal model, oral administration of polyvalent bacterial lysate significantly increased intestinal and pulmonary microbiota diversity, enhanced the abundance of *Bacteroidetes*, *Bacteroidales*, and *Lactobacillus*, inhibited TLR4/NF-κB pathway activation, attenuated pulmonary inflammatory responses, and improved lung function (31).

### 2.3 Feedback regulation of infection on gut microbiota

Respiratory infections themselves can exert significant feedback regulatory effects on the gut microbiota, thereby forming a vicious cycle of infection-dysbiosis. This bidirectional regulatory relationship holds an important position within the theoretical framework of the gut-lung axis. The clinical significance lies in the fact that this bidirectional regulatory relationship suggests that pneumonia-diarrhea comorbidity may form a vicious cycle of infection-dysbiosis-immune disorder-dual infection. Respiratory infections lead to gut microbiota dysbiosis, which in turn, through the gut-lung axis, adversely affects pulmonary immune function and increases the risk of secondary infections. Early identification of gut microbiota dysbiosis and implementation of interventions, such as rational use of microecological preparations and optimization of antibiotic strategies, hold promise for breaking this cycle and improving the clinical prognosis of children with comorbidities.

Evidence from animal experiments indicates that respiratory viral infections can induce characteristic alterations in gut microbiota composition. In a mouse model of H1N1 influenza virus infection, the abundance of the phylum Bacteroidetes significantly increased following infection, accompanied by a corresponding decrease in Firmicutes, suggesting a marked shift in microbial community structure (29). H7N9 influenza virus infection led to a significant reduction in gut microbiota *α*-diversity and decreased microbial community stability, manifested as a notable decrease in beneficial commensal bacteria such as *Eubacterium*, *Ruminococcus*, *Bifidobacterium*, and *Roseburia*. Respiratory syncytial virus infection in mice also resulted in significant changes in gut microbiota structure, further confirming the remote regulatory effect of respiratory viral infections on the intestinal microecology (32–34).

Clinical research data further validate this feedback regulatory effect. Serum testing in patients with community-acquired pneumonia revealed significantly elevated levels of lipopolysaccharide, TNF-α, and IL-6, suggesting a correlation between gut microbiota dysbiosis and increased lipopolysaccharide translocation into the bloodstream, as well as systemic inflammatory activation. Functional prediction analysis indicated that the gut microbiota of patients with pneumonia exhibited enrichment in pathways related to carbohydrate metabolism and bacterial infection. In children with COVID-19, gut microbiota evenness was significantly higher compared to healthy controls, with increased relative abundance of *Bacteroidetes* and *Firmicutes*, decreased *Proteobacteria*, higher relative abundance of opportunistic pathogens, and reduced abundance of beneficial commensal bacteria, suggesting that SARS-CoV-2 infection can also lead to intestinal microecological imbalance (35, 36).

**Figure 1:**
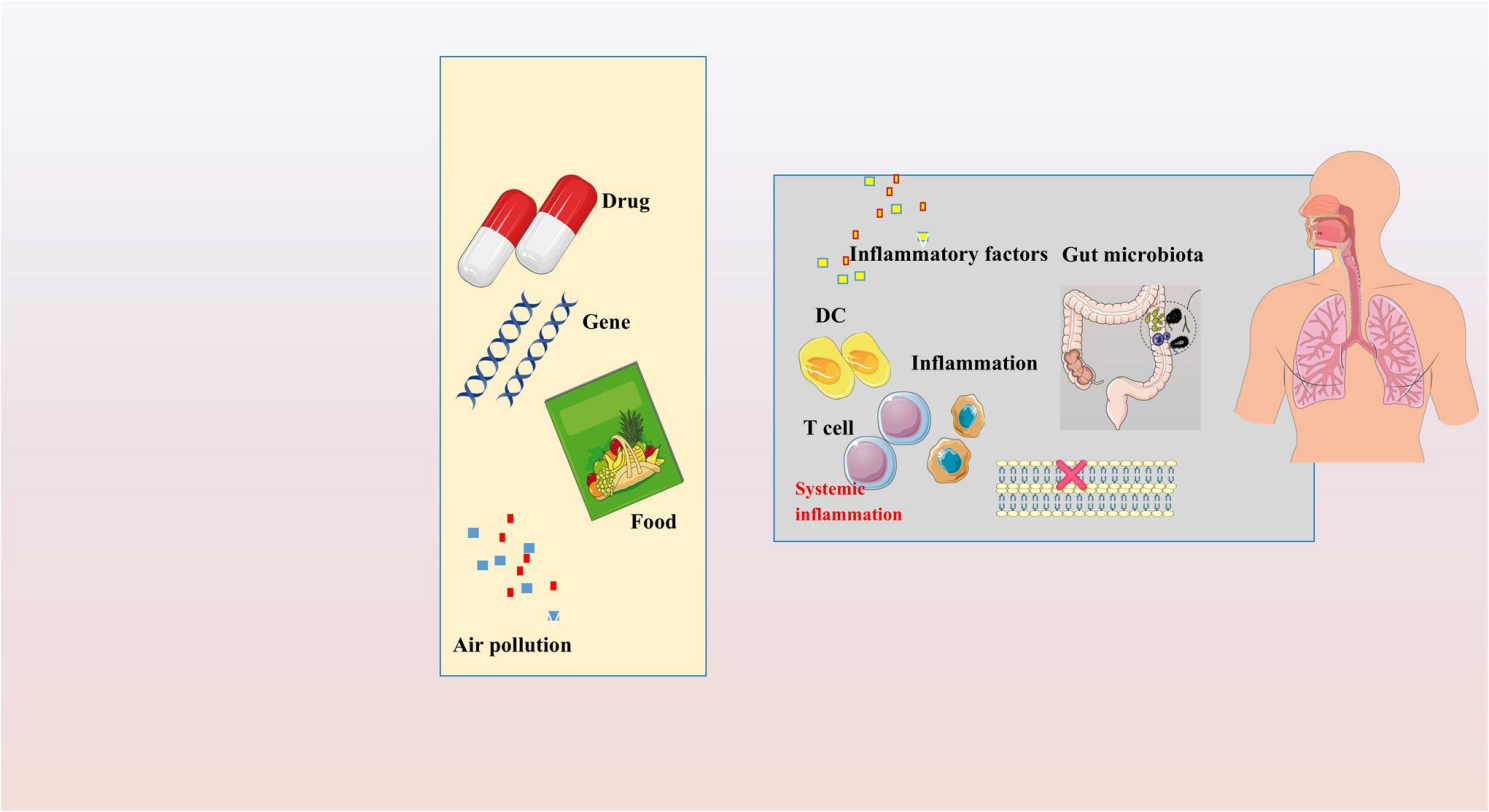
Bidirectional interactions of the gut-lung axis in pediatric pneumonia-diarrhea comorbidity.

At the mechanistic level, the feedback regulation of infection on gut microbiota is primarily achieved through the following four pathways. First, the type I interferon response induced by viral infection directly affects the intestinal microenvironment by regulating gene expression in intestinal epithelial cells. Second, reduced food intake during infection leads to altered substrate availability in the gut, affecting microbial metabolic activities and growth conditions. Thirdly, systemic inflammatory responses indirectly disrupt intestinal homeostasis through cytokine networks such as TNF-α, IL-6, and IL-1β, increasing intestinal permeability. Fourth, the use of antibiotics in clinical treatment further exacerbates microbial imbalance, promoting the colonization of opportunistic pathogens in Figure 1.

## 3 Mechanisms and manifestations of gut microbiota dysbiosis

### 3.1 Decline in microbial diversity and structural alterations

The decline in gut microbiota diversity and structural alterations represent the most core manifestations of microecological imbalance in pneumonia and pneumonia-diarrhea comorbidity. This imbalance can manifest as a decrease in α-diversity indices or as changes in the relative abundance of specific microbial phyla, and is closely associated with disease onset and progression. In the context of pneumonia and pneumonia-diarrhea comorbidity, the gut microbiota exhibits an imbalance characterized by decreased diversity, reduction in beneficial commensal bacteria, and increase in opportunistic pathogens as core features. Such structural changes in the microbiota may be either secondary phenomena following infection or may contribute to disease progression, forming a critical component of the infection-dysbiosis cycle. Evidence from animal experiments has provided direct evidence for respiratory infection-induced alterations in gut microbiota structure. A study using K18-hACE2 transgenic mice infected with SARS-CoV-2 demonstrated that the high-dose virus challenge group exhibited significantly reduced Shannon index in cecal microbiota, with microbial community structure showing marked differences from the control group (37–39). Specifically, the abundance of *Firmicutes* significantly decreased, particularly *Lachnospiraceae* and *Oscillospiraceae*; while *Verrucomicrobia* abundance significantly increased, with *Akkermansiaceae* being enriched at the peak of infection. Similarly, a *Streptococcus pneumoniae* colonization mouse model confirmed that pathogen introduction led to a significant temporary decline in respiratory microbiota diversity, which gradually recovered to baseline levels as the bacteria were cleared (39). These studies reveal that respiratory viral infections or bacterial colonization can induce characteristic changes in gut microbiota structure through remote regulatory mechanisms.

Clinical research evidence further confirms the characteristics of decreased diversity and structural alterations in the gut microbiota of patients with pneumonia. A prospective case-control study of patients with community-acquired pneumonia showed that CAP patients had significantly reduced gut microbiota α-diversity, with β-diversity showing significant differences at the OTU level and across all taxonomic levels (40, 41). Specifically, short-chain fatty acid-producing genera such as *Blautia* and *Agathobacter* were significantly decreased, while opportunistic pathogenic genera including *Gemmiger*, *Enterocloster*, and *Thomasclavelia* were significantly enriched. Functional prediction analysis indicated that the gut microbiota of CAP patients exhibited enrichment in pathways related to carbohydrate metabolism and bacterial infection (41). Another study on antibiotic treatment in children with community-acquired pneumonia using 16S rRNA gene sequencing analysis found that 29% of the children developed antibiotic-associated diarrhea. Significant differences in gut microbiota composition were observed between the diarrhea group and the non-diarrhea group. Higher baseline abundance of two *Bacteroides* species was associated with lower diarrhea risk, while higher abundance of *Lachnospiraceae* and amino acid biosynthesis pathways was associated with increased diarrhea risk (42). Children in the diarrhea group experienced persistent microbiota imbalance.

The association between microbiota structural changes and clinical prognosis has been confirmed by multiple studies. A multi-site longitudinal microbiome study of patients with acute respiratory failure revealed progressive dysbiosis in oral, lung, and gut microbiota, characterized by decreased microbial diversity, reduction in beneficial anaerobic bacteria, and increase in pathogenic bacteria (43). Interestingly, unsupervised clustering based on lung microbiota diversity and composition independently predicted patient survival outcomes, with predictive efficacy superior to traditional clinical indicators and host response markers. Studies on childhood respiratory infections have also found that lower nasal microbiota α-diversity and reduced *Prevotella* abundance at 2 months of age were significantly associated with a history of early lower respiratory tract infections (44).

A prospective cohort study in rural Malawi further revealed the bidirectional relationship between infection and microbiota diversity. Study has found that diarrheal symptoms during 11–12 months of age predicted subsequent decreases in microbiota-for-age z-score, while respiratory symptoms were associated with increased Shannon index. Higher microbiota maturity and diversity at 6 months of age predicted a reduced incidence of fever over the subsequent 6 months (45). This finding suggests that early microbiota status has significant predictive value for subsequent infection susceptibility. Studies on the microbiota characteristics of children with pneumonia and secondary diarrhea have provided more targeted evidence (45). Analysis of 50 children with pneumonia and secondary antibiotic-associated diarrhea showed that, compared to children with pneumonia alone without diarrhea, children with comorbidity had significantly reduced fecal *Bifidobacterium* counts, significantly increased *Escherichia coli* counts, and a markedly decreased *Bifidobacterium*-to-*Escherichia coli* ratio (46). This structural change in microbiota was closely associated with abnormalities in intestinal barrier function indicators.

### 3.2 Abnormal short-chain fatty acid metabolism

SCFAs, primarily including acetate, propionate, and butyrate, are crucial metabolites produced by gut microbiota through the fermentation of dietary fiber. They play a central role in maintaining intestinal barrier function and regulating local and systemic immune responses. In the context of pneumonia and pneumonia-diarrhea comorbidity, SCFA metabolic abnormalities manifest as a decline in the abundance of SCFA-producing bacteria, reduced SCFA levels, and impaired SCFA-mediated immunomodulatory functions.

Animal experiments have provided direct evidence for SCFA metabolic abnormalities and their impact on pulmonary immunity. A study by Hu et al. using a mouse model of influenza virus infection demonstrated that the infected group exhibited a significant reduction in the relative abundance of acetate-producing bacteria including *Bacteroides*, *Bifidobacterium*, and *Akkermansia* in the gut, accompanied by decreased acetate levels in both intestinal contents and serum (47, 48). Paired feeding experiments confirmed that these changes were partly attributable to reduced substrate supply due to decreased food intake during infection. Antibiotic-induced depletion of gut microbiota exacerbated influenza virus-induced lung inflammatory injury, whereas fecal microbiota transplantation restored the impaired pulmonary barrier function. Mechanistic studies revealed that acetate pretreatment partially restored influenza virus-induced airway epithelial barrier function by activating G protein-coupled receptor 43 (GPR43), as evidenced by increased transepithelial electrical resistance, decreased permeability, and reduced release of pro-inflammatory cytokines (49). This protective effect was absent in GPR43-knockdown bronchial epithelial cells, suggesting that the acetate-GPR43 signaling axis plays a critical role in maintaining airway epithelial barrier integrity (50, 51).

The impact of butyrate on pulmonary function was further observed in a mouse model of sepsis-associated lung injury. Sodium butyrate administration following cecal ligation and puncture was associated with improved survival rates, enhanced oxygenation index, and attenuated lung histopathological damage (52). Mice receiving sodium butyrate showed reduced lung wet-to-dry ratio, decreased total protein concentration in bronchoalveolar lavage fluid, and altered cytokine profiles, with reduced pro-inflammatory and elevated anti-inflammatory cytokines. Enhanced expression of intercellular junction proteins such as occludin and ZO-1 in both intestinal and lung tissues, as well as increased proportion of CD4 + Foxp3+ regulatory T cells and CD4+/CD8+ T cell ratio, were observed (53). Research on prenatal antibiotic exposure and offspring asthma risk further revealed that maternal antibiotic exposure during pregnancy significantly reduced butyrate levels in the gut microbiota of neonatal mice, which was associated with elevated frequency and activity of type 2 innate lymphoid cells (ILC2s) in lung tissue and downregulation of type I interferon signaling pathways, correlating with increased susceptibility to allergic airway inflammation in adult offspring (54, 55). Butyrate supplementation was associated with restored type I interferon signaling through GPR41 activation, reduced ILC2 proliferation and IL-13 release, and improved asthma phenotype in offspring (56). Clinical studies have further confirmed the association between SCFA metabolic abnormalities and pneumonia. A metagenomic combined with metabolomic study involving 32 patients with community-acquired pneumonia and 36 healthy controls revealed that patients with pneumonia exhibited significant alterations in gut microbiota composition, with markedly reduced abundance of SCFA-producing genera such as *Faecalibacterium*, *Ruminococcus*, and *Eubacterium*, as well as key butyrate-producing species including *Faecalibacterium prausnitzii*, *Eubacterium rectale*, and *Bifidobacterium adolescentis* (57). Metabolomic analysis further confirmed a decreasing trend in fecal SCFA levels in patients with pneumonia, along with significantly reduced levels of secondary bile acids. Correlation analysis showed positive associations between *F. prausnitzii*, *B. adolescentis*, and *E. rectale* with ursodeoxycholic acid, suggesting synergistic dysregulation between SCFA and bile acid metabolism. Intervention studies on SCFAs in childhood pneumonia have provided important evidence for causal validation. A randomized, double-blind, placebo-controlled trial in healthy children evaluated the preventive effects of *Bifidobacterium longum* subsp. infantis YLGB-1496 on common pediatric diseases. Results showed that the incidence of upper respiratory tract infections was significantly lower in the probiotic group compared to the placebo group, with significantly reduced rates of bronchopneumonia and eczema as well (58). Microbiota analysis revealed significantly increased relative abundance of *Bifidobacterium* and decreased abundance of the opportunistic pathogen *Bacteroides* in the probiotic group. The probiotic group showed decreased fecal levels of pro-inflammatory factors and increased levels of immunoglobulins including IgA, IgG, and IgM, and SCFAs including butyrate and total SCFAs, directly confirming the preventive value of modulating the gut microbiota-SCFA axis against respiratory infections (58).

Epidemiological data indicate that reduced levels of the three main SCFAs in early life are associated with the development of childhood atopic dermatitis, wheezing, and IgE-mediated food allergies. Cohort studies have shown that farm-exposed infants exhibit significantly higher gut microbiota diversity in the first year of life, and higher microbiota diversity correlates with lower asthma risk, suggesting that the protective effect may be mediated through the microbiota-SCFAs axis. Synthesizing the above evidence, the pathophysiological significance of SCFA metabolic abnormalities in pneumonia and pneumonia-diarrhea comorbidity can be summarized as follows. First, respiratory infections can decrease the abundance of SCFA-producing bacteria through mechanisms including reduced food intake and systemic inflammatory responses, forming a cycle of infection-dysbiosis (59, 60). Secondly, decreased SCFA levels in the intestine and circulation are associated with impaired SCFA-related biological functions. Thirdly, insufficient SCFAs correlate with reduced expression of tight junction proteins in intestinal and airway epithelia, increased intestinal permeability, and translocation of bacterial products into the circulation. Fourth, SCFA deficiency is associated with reduced regulatory T cell differentiation and excessive ILC2 activation, which correlate with disrupted immune homeostasis and increased airway hyperresponsiveness.The potential therapeutic value lies in the fact that supplementation with SCFAs or modulation of SCFA-producing microbiota can partially reverse the aforementioned pathological changes, improving pulmonary barrier function and immune regulation, thereby providing new intervention targets for the prevention and treatment of pneumonia and its comorbidities. In conclusion, in the context of pneumonia and pneumonia-diarrhea comorbidity, SCFA metabolic abnormalities manifest as reduced abundance of SCFA-producing bacteria, decreased SCFA levels, and impaired barrier function and immunomodulation mediated by these metabolites. This metabolic dysregulation is not only a secondary phenomenon following infection but can also reciprocally influence pulmonary immune status through the gut-lung axis, constituting a critical link in disease progression. Intervention strategies targeting SCFA metabolism, including dietary fiber supplementation, probiotic modulation, and direct SCFA supplementation, hold broad promise for clinical application.

### 3.3 Increased colonization by opportunistic pathogens

Another core manifestation of gut microbiota dysbiosis is the increased colonization by opportunistic pathogens. In the context of pneumonia and pneumonia-diarrhea comorbidity, the reduction of beneficial commensal bacteria represented by *Bifidobacterium* and *Lactobacillus* disrupts the colonization resistance capacity of the microbiota, creating niche conditions for the proliferation of opportunistic pathogens such as *Clostridium difficile*, *Klebsiella pneumoniae*, and *Enterococcus*, thereby increasing the risk of secondary infections. The increased colonization by opportunistic pathogens is not only a consequence of microbiota dysbiosis but also a driving factor in disease progression. *C. difficile* infection can lead to antibiotic-associated diarrhea, exacerbating intestinal barrier damage; intestinal colonization by drug-resistant pathogens such as *K. pneumoniae* can serve as a source of systemic infection, increasing the risk of secondary bacteremia. Probiotic interventions can inhibit the growth of opportunistic pathogens, and fecal microbiota transplantation can effectively eliminate multidrug-resistant organism colonization, providing effective means to break the vicious cycle of infection-dysbiosis-opportunistic pathogen colonization.

Animal experimental evidence indicates that respiratory infections can lead to the enrichment of intestinal opportunistic pathogens. In a mouse model of influenza A virus infection, significant alterations in gut microbiota composition were observed following infection, with increased relative abundance of opportunistic pathogens (61). Fecal microbiota transplantation experiments confirmed that transplanting microbiota from healthy donors to infected mice could restore intestinal barrier function and inhibit pathogen colonization. Studies using a co-infection model of porcine reproductive and respiratory syndrome virus and porcine circovirus type 2 revealed that gut microbiota diversity was closely associated with clinical outcomes, pigs exhibiting high growth rates, low viral replication levels, and mild pneumonia severity showed higher microbiota diversity and increased abundance of *Ruminococcaceae*, while *Methanobacteriaceae* abundance was decreased (62). This finding suggests that microbiota composition plays an important role in determining host susceptibility to respiratory pathogens. A mouse model of *Streptococcus pneumoniae* pulmonary infection further confirmed that gut microbiota has a protective effect against respiratory pathogens. Antibiotic pretreatment depleting gut microbiota significantly increased mouse susceptibility to *S. pneumoniae* infection, while fecal microbiota transplantation restored this protective effect, suggesting that an intact gut microbiota structure is crucial for maintaining colonization resistance and preventing pathogen dissemination (62).

Clinical research evidence further confirms the characteristic increase in intestinal opportunistic pathogen colonization in patients with pneumonia. A retrospective study of elderly patients with severe pneumonia analyzed gut microbiota changes through amplicon sequencing. Results showed that during antibiotic treatment, patients in the control group exhibited significant increases in opportunistic pathogens at the genus level, including *Citrobacter* and *Massilia* (63). In contrast, in the probiotic intervention group, the growth of opportunistic pathogens was inhibited, while the abundance of butyrate-producing bacteria increased. This finding directly confirms the causal relationship between antibiotic exposure and opportunistic pathogen colonization, as well as the protective value of probiotic intervention (64).

C. *difficile* is the most important pathogen in antibiotic-associated diarrhea, and its infection is closely related to microbiota dysbiosis. Systematic reviews have shown that risk factors for *C. difficil*e infection include prolonged hospitalization, antibiotic exposure, particularly clindamycin, cephalosporins, and penicillins, proton pump inhibitor use, invasive mechanical ventilation, immunosuppression, and underlying comorbidities. In the pediatric population, the incidence of community-acquired *C. difficile* infection is increasing, with clindamycin exposure significantly associated with infection risk, and amoxicillin-clavulanate and cephalosporins also identified as independent risk factors (65). The asymptomatic carriage rate of *C. difficile* in infants is as high as 84%, but clinical infection is rare, possibly related to breast milk immunoglobulins blocking toxin binding, immature intestinal mucosa, and protective microbiota composition. The problem of multidrug-resistant *K. pneumoniae* is particularly prominent in patients with severe pneumonia (66). A case report of a 95-year-old patient with severe pneumonia secondary to COVID-19 detailed the diagnosis and treatment process of pandrug-resistant K. pneumoniae infection. During hospitalization, the patient experienced recurrent infections with ESBL-positive pandrug-resistant *K. pneumoniae*, which were unresponsive to conventional anti-infective therapy (66). Gut microbiota testing revealed significantly reduced microbial diversity, increased abundance of *Klebsiella*, and marked depletion of beneficial bacteria such as *Prevotella*, *Phascolarctobacterium*, *Ruminococcus*, *Acidaminococcus*,and *Lactobacillus*. Following fecal microbiota transplantation via nasojejunal tube, the patient's diarrhea ceased, airway secretions decreased significantly, and microbiota analysis showed increased beneficial bacteria abundance and decreased *Klebsiella* relative abundance, ultimately leading to successful hospital discharge (66–68). This case directly confirms the therapeutic value of fecal microbiota transplantation in inhibiting opportunistic pathogen colonization and restoring microbial balance.

Analysis of gut microbiota in children with respiratory syncytial virus infection showed that severely infected children had increased relative abundance of Clostridium and Lactobacillus, while Moraxellaceae bacteria decreased. Gut microbiota in children with COVID-19 also showed characteristic changes, significantly increased microbial evenness, increased *Bacteroidetes* and *Firmicutes*, decreased *Proteobacteria*, higher relative abundance of opportunistic pathogens including *Pseudomonas*, *Herbaspirillum*, and *Burkholderia*, and decreased beneficial commensal bacteria (69). A systematic review and meta-analysis integrating studies on gut microbiota changes in patients with respiratory infections including 11 studies covering SARS-CoV-2 infection, influenza, tuberculosis, and community-acquired pneumonia showed that compared to healthy controls, respiratory infection patients had a mean reduction in gut microbiota Shannon index of 1.45 units, decreased abundance of *Lachnospiraceae*, *Ruminococcaceae*, and *Ruminococcus*, and enrichment of *Enterococcus*. This finding confirms, at the level of evidence-based medicine, that the increase in opportunistic pathogen colonization caused by respiratory infections is a universal phenomenon (70).

At the mechanistic level, the mechanisms underlying increased opportunistic pathogen colonization can be summarized as follows. First, antibiotic exposure directly kills sensitive commensal bacteria, creating ecological niches for drug-resistant pathogens. Secondly, decreased microbial diversity leads to loss of colonization resistance function, increasing the availability of nutrients and adhesion sites for pathogens. Thirdly, reduced microbial metabolites, such as SCFAs, weaken their inhibitory effects on pathogen growth. Fourth, systemic inflammatory responses increase intestinal permeability, promoting translocation of pathogens and their products, further activating inflammatory cascades.

## 4 Molecular mechanisms of immune dysregulation

### 4.1 Th1/Th2 imbalance and dysregulation of the inflammatory cytokine network

The Th1/Th2 balance serves as a core regulatory node of the immune response. Th1 cells primarily secrete interleukin-2, interferon-γ, and tumor necrosis factor-β, mediating cellular immune responses against intracellular pathogens. Th2 cells mainly secrete IL-4, IL-5, IL-6, IL-10, and IL-13, mediating humoral immune responses closely associated with extracellular pathogen clearance and allergic reactions. In the context of pneumonia and pneumonia-diarrhea comorbidity, the Th1/Th2 balance undergoes significant deviation, accompanied by systemic dysregulation of the inflammatory cytokine network, constituting the core molecular basis of immune dysregulation.

*Staphylococcus aureus*, a common pathogen in pediatric respiratory infections, induces immune responses with mixed characteristics. Studies have demonstrated that S. aureus-derived extracellular vesicles can induce neutrophilic pulmonary inflammation by simultaneously activating Th1 and Th17 cell responses (71, 72). This finding suggests that respiratory infections trigger complex T-cell subset responses rather than Th1 or Th2 polarization. Correspondingly, research on Escherichia coli outer membrane vesicles has shown that they mediate protective immunity through Th1 and Th17 cell responses, indicating that enteric pathogens tend to induce Th1-type cellular immune responses. Studies using respiratory syncytial virus mouse infection models have further elucidated the association between Th1/Th2 imbalance and disease severity. RSV infection leads to predominant Th2-type cytokine responses, accompanied by relative deficiency of Th1-type cytokines (72). This imbalance is closely associated with airway hyperresponsiveness and mucus hypersecretion. Th2-type immune bias can be exacerbated by gut microbiota dysbiosis induced by antibiotic exposure during pregnancy or early life, highlighting the critical role of the microbiota-immune axis in regulating Th1/Th2 balance. A case-control study systematically evaluated changes in Th1/Th2 cytokine profiles in children with severe bacterial infections, involving 30 children with infection and 30 healthy controls (73). Some researchers have discovered that compared to healthy controls, children with infection exhibited significantly elevated peripheral blood concentrations of IL-2, IFN-γ, and IL-4, indicating activation of both Th1 and Th2 pathways during infection. However, concentrations of TNF-α, IL-5, and IL-6 were significantly lower than those in healthy controls, suggesting complex reprogramming of the inflammatory cytokine network (74). More critically, the type of infection determined the directionality of Th1/Th2 polarization. Children with gastrointestinal infections showed significantly elevated TNF-α concentrations, while Th2-type cytokines remained below detection thresholds, exhibiting Th1-predominant response characteristics. In contrast, children with respiratory infections demonstrated significantly elevated Th2-type cytokines, accompanied by increasing trends in Th1-type cytokine, presenting a mixed Th1/Th2 response pattern. These findings indicate that children with pneumonia exhibit dual activation of Th1/Th2 pathways, while gastrointestinal infections alone tend toward Th1-predominant responses.

Pathogen detection further revealed associations between infectious agents and immune response patterns. In the study, 5 of 9 children with gastrointestinal infections tested positive for *E. coli* antibodies, including 3 cases of enterohemorrhagic *E. coli* and 2 cases of enteropathogenic *E. coli*. Among 21 children with respiratory infections, 14 tested positive for S. aureus antibodies, and 4 tested positive for *Klebsiella pneumoniae* antibodies (73). S. aureus, as a common pathogen in pediatric respiratory infections, induces mixed Th1/Th2 responses consistent with previous studies, this pathogen can simultaneously activate cellular and humoral immune pathways through multiple virulence factors.

### 4.2 Impairment of mucosal barrier function

Impairment of mucosal barrier function represents another core mechanism of immune dysregulation in pneumonia and pneumonia-diarrhea comorbidity. The intestinal mucosal barrier is composed of the mechanical barrier including intestinal epithelial cells and tight junctions, biological barrier, chemical barrier, and immune barrier. Its integrity is crucial for preventing bacterial and toxin translocation and maintaining local and systemic immune homeostasis. In the comorbid state, respiratory infections can remotely induce intestinal barrier dysfunction through the gut-lung axis, while inflammatory mediators released after intestinal barrier damage can in turn exacerbate pulmonary inflammation, forming a vicious cycle.

Animal experimental evidence provides direct support for respiratory infection-induced intestinal barrier dysfunction. Studies using a respiratory syncytial virus mouse infection model have shown that viral infection not only causes pulmonary inflammation but also remotely induces intestinal barrier dysfunction (75). Following RSV infection, tight junction protein expression in mouse intestinal tissues was significantly downregulated, intestinal permeability increased, and serum D-lactate and endotoxin levels elevated (76). This intestinal barrier damage was closely associated with alterations in gut microbiota structure and Th2-type immune bias. The direct damaging effect of respiratory viral infection on the airway epithelial barrier has also been well-documented. A study by Liu et al. demonstrated that following RSV infection of human airway epithelial cells, epidermal growth factor receptor phosphorylation levels significantly increased, tight junction proteins ZO-1 and occludin were markedly downregulated, cell barrier function was impaired, transepithelial electrical resistance decreased, and permeability increased (75).

Concurrently, mucin MUC5AC expression was significantly upregulated, suggesting synergistic effects between abnormal mucus secretion and barrier dysfunction. Further mechanistic studies revealed that RSV disrupts epithelial barrier integrity and promotes viral replication by activating the EGFR signaling pathway and inhibiting type I interferon responses (77). A mouse model of *Streptococcus pneumoniae* pulmonary infection also confirmed that respiratory bacterial infection can induce intestinal barrier dysfunction (78). Following infection, tight junction protein expression in mouse intestinal tissues was downregulated, intestinal permeability increased, and serum endotoxin and D-lactate levels elevated, with these changes positively correlated with the severity of pulmonary inflammation. Antibiotic pretreatment depleting gut microbiota significantly exacerbated this effect, suggesting that an intact gut microbiota is essential for maintaining barrier function (79).

Clinical research evidence further confirms significant intestinal barrier dysfunction in children with pneumonia. Hand, foot, and mouth disease (HFMD), an infectious disease caused by enteroviruses that frequently involves both the respiratory and digestive tracts, serves as an ideal model for studying mucosal barrier function. A study by Liu et al. involving 470 children with varying severity of HFMD showed that plasma D-lactate levels were significantly elevated in the study group compared to healthy controls. Plasma D-lactate levels were positively correlated with disease severity, the critical group had significantly higher levels than the severe group, which in turn had significantly higher levels than the moderate group and control group, with statistically significant differences between groups. This finding suggests that D-lactate, as a sensitive indicator of intestinal permeability, can early reflect the degree of intestinal mucosal barrier injury.

Plasma diamine oxidase (DAO), a cytosolic enzyme in intestinal mucosal epithelial cells, reflects intestinal mucosal integrity through changes in its activity. In the aforementioned study, although the difference in DAO levels between the study and control groups did not reach statistical significance, DAO levels in the critical group showed an increasing trend, suggesting possible intestinal mucosal cell damage in severe infection states. Plasma endotoxin, a component of Gram-negative bacterial cell walls, reflects intestinal barrier function and the degree of bacterial translocation through its levels in the bloodstream. Endotoxin levels in the critical group were higher than in other groups, although intergroup differences did not reach statistical significance (80). Studies on neonatal necrotizing enterocolitis provide further support for the clinical significance of intestinal barrier function indicators. A study by Zhang et al. on 78 children with NEC showed that serum D-lactate, DAO, and endotoxin levels were significantly higher in NEC children than in normal neonates, and these indicators progressively increased with advancing NEC Bell stages, with statistically significant differences between all groups. This finding confirms that D-lactate, DAO, and endotoxin are sensitive indicators reflecting intestinal mucosal barrier injury, with levels closely correlated with disease severity (80).

The association between intestinal barrier function and gut microbiota has been confirmed in clinical studies. Research on elderly patients with severe pneumonia showed that during antibiotic treatment, the abundance of intestinal opportunistic pathogens significantly increased, while probiotic intervention inhibited the growth of opportunistic pathogens and increased the abundance of butyrate-producing bacteria. Butyrate, as the primary energy source for intestinal epithelial cells, plays a key role in maintaining tight junction protein expression and intestinal barrier integrity. Studies on ventilator-associated diarrhea provide important clues regarding intestinal barrier damage in critically ill patients (81). A retrospective analysis by Que et al. of 56 mechanically ventilated patients showed that 33% of patients developed diarrhea, lasting an average of 13 days. Fecal examination revealed abundant spores in 8 patients, hyphae in 3 patients, and dysbiosis in 4 patients, decreased bacilli, increased cocci, with *Enterococcus faecalis* detected in 3 cases. Logistic regression analysis confirmed a significant correlation between ventilator-associated pneumonia and diarrhea during mechanical ventilation, suggesting that pulmonary infection is an important driver of intestinal barrier dysfunction and secondary diarrhea (82).

**Figure 2:**
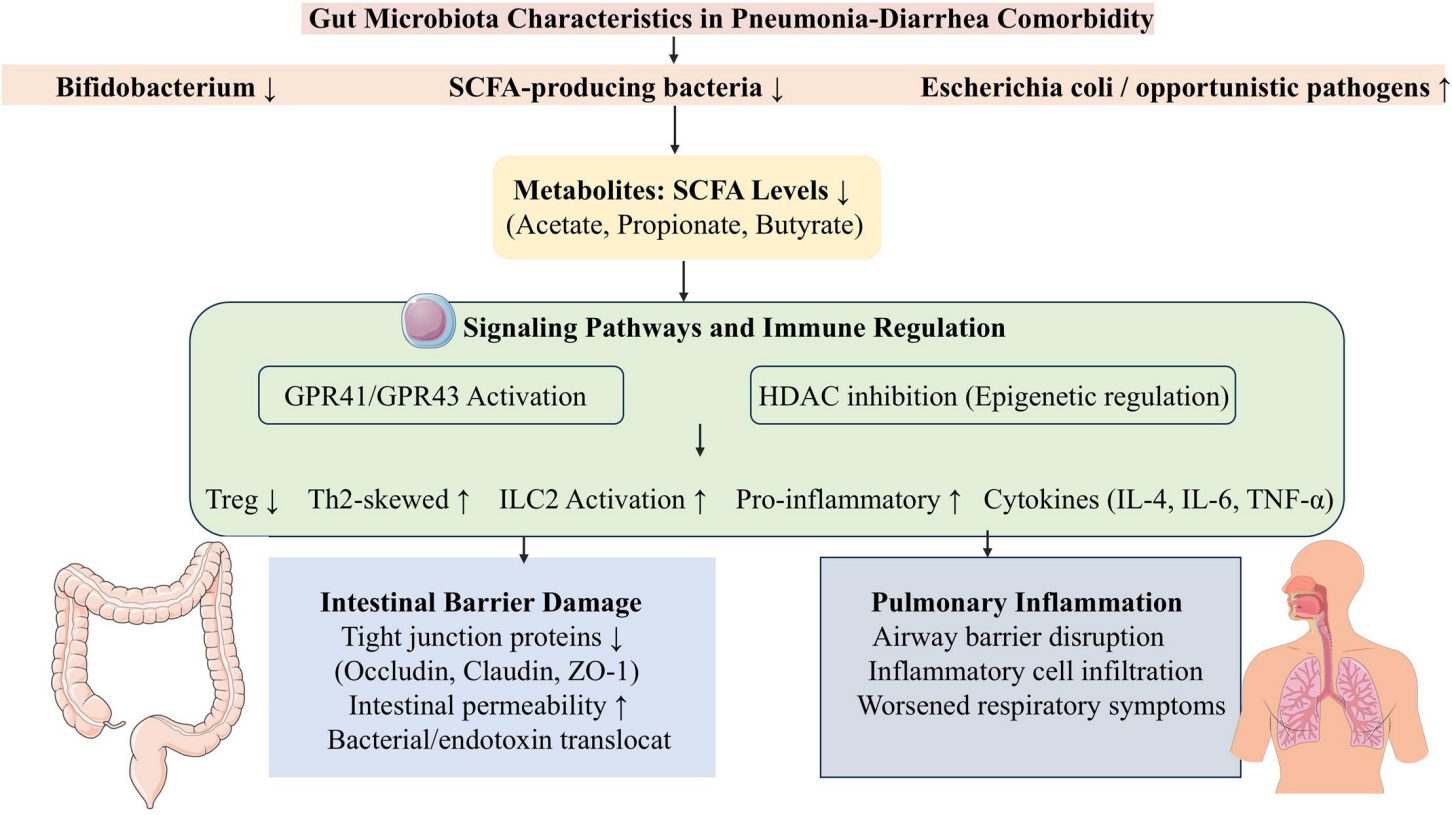
Detailed molecular pathways of gut-lung axis dysregulation in children with pneumonia-diarrhea comorbidity.

In summary, respiratory infections release pro-inflammatory cytokines through systemic inflammatory responses, which can remotely downregulate intestinal epithelial tight junction protein expression and increase intestinal permeability. Viral infections directly inhibit airway epithelial tight junction protein expression and disrupt airway barrier function by activating the EGFR signaling pathway. Gut microbiota dysbiosis leads to reduced butyrate-producing bacteria; butyrate deficiency, as butyrate is the primary energy source for intestinal epithelial cells and a regulator of tight junction protein expression, can exacerbate barrier dysfunction. Furthermore, increased intestinal permeability allows bacterial products to enter the bloodstream, activating systemic inflammatory responses that further aggravate pulmonary inflammation, forming a vicious cycle of infection-dysbiosis-barrier injury-exacerbated inflammation in the Figure 2.

## 5 Clinical laboratory indicators and their significance

### 5.1 Combined detection of inflammatory markers

The combined detection of inflammatory markers holds significant clinical value in the diagnosis and severity assessment of pediatric pneumonia and pneumonia-diarrhea comorbidity. C-reactive protein (CRP), serum amyloid A (SAA), IL-6, procalcitonin (PCT), and white blood cell count (WBC), as routine clinical inflammatory indicators, provide objective evidence for early infection recognition, pathogen type differentiation, and disease severity evaluation through their individual or combined application.

International studies have provided substantial evidence for the value of combined inflammatory marker detection. A study conducted at Shanghai Children's Medical Center involving 206 hospitalized children with community-acquired pneumonia systematically evaluated the efficacy of SAA and CRP in differentiating viral from bacterial pneumonia. Results showed that CRP and SAA levels were significantly higher in the bacterial pneumonia group compared to the viral pneumonia group. ROC curve analysis revealed that the area under the curve for CRP and SAA was 0.84 and 0.85, respectively. Combining SAA and CRP detection with clinical symptoms increased diagnostic specificity to 93.2% and 97.3%, providing reliable tools for clinical differential diagnosis. The value of inflammatory markers in pathogen discrimination has been validated in international research. An Indonesian study of 155 children with community-acquired pneumonia demonstrated that white blood cell counts, absolute neutrophil counts, neutrophil-lymphocyte ratio, CRP, and PCT were significantly higher in bacterial and mixed infection cases compared to pure viral infections. Multivariate logistic regression analysis identified as one of the strongest predictors of bacterial or mixed infection (83). This finding suggests that combining clinical features with inflammatory markers can effectively differentiate bacterial from viral pneumonia, providing evidence-based guidance for rational antibiotic use. Combined host-response biomarkers have shown unique value in atypical pathogen infections. A multicenter study by Papan et al. involving 80 children with community-acquired pneumonia evaluated the diagnostic performance of the BV score, which incorporates TRAIL, IP-10, and CRP. Results showed that the BV score effectively differentiated bacterial from viral infections and exhibited unique diagnostic characteristics in children with *Mycoplasma pneumoniae* infection, offering new insights for early recognition of atypical pathogen infections (83).

The prognostic significance of dynamic monitoring has been elucidated in international studies. A US multicenter prospective cohort study of pediatric sepsis revealed that PCT, CRP, and IL-6 levels within 24 h of admission were significantly positively correlated with the degree of organ dysfunction. Dynamic monitoring demonstrated that the magnitude of PCT decrease within 72 h was significantly associated with 28-day mortality and length of hospital stay, with children showing a decrease having significantly better prognosis than those with a smaller decrease. Prediction models based on inflammatory marker changes at admission and 72 h showed good discriminative ability for identifying children requiring intensive care. Age-specific combination strategies warrant attention. A German multicenter study of neonatal sepsis compared the diagnostic value of IL-6, IL-8, PCT, and CRP. Results showed that IL-6 and IL-8 were optimal indicators for early diagnosis, while PCT demonstrated the best diagnostic performance during the 24 to 48 h-window. Based on AUC analysis, combined IL-6 and PCT detection significantly improved diagnostic accuracy, particularly in very low birth weight infant. This suggests that optimal combinations of inflammatory markers should consider differences in patient age and physiological status.

Although predictive studies on pneumonia-associated diarrhea predominantly originate from Chinese populations, international pediatric pneumonia cohort studies have confirmed that diarrhea is a common complication of childhood pneumonia and is associated with elevated inflammatory marker levels. A US multicenter study of children hospitalized with pneumonia showed that CRP levels were significantly higher in children with gastrointestinal symptoms compared to those without, suggesting that the degree of systemic inflammatory response is correlated with the risk of gastrointestinal involvement.

### 5.2 Nutritional and immune status indicators

Nutritional status is closely related to immune function, collectively constituting a critical line of defense against infections in children. In the context of pneumonia and pneumonia-diarrhea comorbidity, nutritional and immune status indicators not only reflect the baseline health status of affected children but also serve as key biomarkers for predicting infection susceptibility and disease severity. Serum levels of prealbumin, zinc, ferritin, and other micronutrients, along with cellular immune function indicators, provide important evidence for early identification and intervention in comorbidities. In clinical practice, comprehensive assessment of nutritional and immune status indicators should be performed to identify high-risk children early and implement precision nutritional interventions, thereby breaking the malnutrition-infection vicious cycle.

The association between nutritional status and infection risk has been confirmed by multiple international studies. Malnutrition is a major risk factor for childhood pneumonia and diarrhea, while infection itself further impairs nutritional status, forming a vicious cycle. Research emphasizes that diarrheal episodes increase pneumonia susceptibility in malnourished children, suggesting a nutrition-mediated interaction between gastrointestinal and respiratory infections. Breastfeeding and micronutrient supplementation have been proven effective measures for preventing and controlling diarrhea and pneumonia.

Cellular immune function serves as a functional indicator of nutritional status with significant predictive value. A 10-month prospective study conducted by Shell-Duncan et al. in nomadic communities of northern Kenya, including children aged 6 months to 10 years, assessed cellular immune function through delayed-type hypersensitivity and analyzed its association with the incidence of diarrhea and acute respiratory infections. Results showed that children unresponsive to delayed-type hypersensitivity tests had an average diarrhea incidence 20% higher than immunocompetent children (84, 85). During the rainy season, both nutritional status and delayed-type hypersensitivity significantly predicted acute respiratory infection incidence, but when both were included in the analysis, only delayed-type hypersensitivity maintained significant predictive value unresponsive children had respiratory infection incidence 34% higher than immunocompetent children. This finding indicates that cellular immune status is a sensitive predictor of gastrointestinal and respiratory infections, and the impact of nutritional status on respiratory infections may be partially mediated through cellular immune function (85).

A community-based longitudinal study in rural Bangladesh involving 696 children aged 0–59 months further validated this association. Children were prospectively followed for one year, with respiratory infection symptom data collected every 4 days, anthropometric measurements monthly, and cellular immune function assessed every 3 months through multi-antigen skin testing. Results showed an acute upper respiratory infection incidence of 5.3 episodes per person-year. In regression models, children with wasting malnutrition had a 16% increased risk of upper respiratory infection, while unresponsive children had a 20% increased risk compared to immunocompetent children (86). Wasting malnutrition and impaired cellular immune function are independent risk factors for childhood upper respiratory infections, whereas stunting showed no significant association. Micronutrient deficiency is an important mechanism of immune impairment (87, 88). Research by Palmer et al. indicates that approximately 5 million children die annually from preventable causes, including respiratory infections, diarrhea, and malaria, with about half of these deaths attributable to malnutrition, including micronutrient deficiencies. The impact of infection on micronutrient status is well-established, pathogen-induced inflammatory responses cause anorexia, while pathogens themselves and immune responses can alter nutrient absorption and lead to nutrient loss. Vitamins A and D, iron, zinc, and selenium play key roles in the immune system by regulating host defense at molecular or cellular levels, directly affecting pathogens, or protecting the body from oxidative stress and inflammatory damage (88).

The application of zinc and vitamin A in infection treatment has been extensively studied. Zinc supplementation in acute diarrhea significantly reduces persistent episodes and moderately shortens episode duration; in persistent diarrhea, it reduces treatment failure and mortality. However, zinc supplementation shows no significant efficacy in measles or non-measles pneumonia, and high-dose zinc in severely malnourished children may increase mortality risk. Vitamin A supplementation during measles episodes significantly reduces mortality and may reduce persistent diarrheal episodes in children with acute diarrhea, but shows no benefit in pneumonia. Therefore, high-dose vitamin A is recommended during measles episodes but not for non-measles pneumonia. The direct association between nutritional biomarkers and infection burden was elucidated in the ELICIT study in rural Tanzania. The study included 1,140 children, with fecal samples collected at 6, 12, and 18 months to detect intestinal pathogen carriage, and serum nutritional and inflammatory biomarkers assessed. Results showed median pathogen counts of 2, 3, and 2 at the three time points, with an overall mean pathogen detection count of 7.79 (89). Predictive analysis revealed that each additional pathogen was significantly associated with a 0.03 decrease in length-for-age Z-score and a 0.04 decrease in weight-for-age Z-score at 18 months. Exploring potential mechanisms of growth impairment, the study found that pathogen burden was positively correlated with serum C-reactive protein and FGF21, and negatively correlated with collagen X and IGF-1 associations also present in Shigella-only infections. This finding directly confirms that intestinal pathogen carriage leads to childhood growth impairment through inducing systemic inflammation and suppressing growth factors, providing important evidence for the mechanisms of nutrition-infection interactions (89).

The intervention effects of oral nutritional supplementation have been supported by systematic reviews. A meta-analysis including 14 studies evaluated the impact of oral nutritional supplementation on health outcomes and nutritional biomarkers in malnourished children. Results showed that compared to dietary counseling alone, oral nutritional supplementation reduced upper respiratory infection incidence by 39% in nutritionally at-risk children. This finding suggests that improving nutritional status can enhance immune function, effectively breaking the malnutrition-infection vicious cycle. Prealbumin and zinc as sensitive indicators of nutritional status have been validated in clinical studies. A study at Zagazig University involving 40 children aged 1–5 years with acute lower respiratory infections showed that zinc and prealbumin levels were influenced by children's nutritional status, and improved nutritional status was associated with significantly reduced respiratory infection mortality and morbidity (90, 91). Although no significant differences in zinc and prealbumin levels were observed among children with pneumonia, bronchitis, and bronchiolitis, body mass index and nutritional status grades differed significantly between groups, suggesting that nutritional status is an important determinant of infection type and severity (91).

### 5.3 Intestinal barrier function indicators and microbiota detection

Currently, the clinical value of intestinal barrier function indicators in children with pneumonia-diarrhea comorbidity remains at an exploratory stage, as most available evidence is derived from adult studies or small-sample pediatric studies. Large-scale prospective validation studies are still lacking. Nevertheless, preliminary evidence suggests that serum D-lactate, diamine oxidase, and endotoxin may serve as sensitive markers of intestinal mechanical barrier damage, enabling early identification of intestinal injury. Serum D-lactate, diamine oxidase, and endotoxin serve as sensitive markers of intestinal mechanical barrier damage, enabling early identification of intestinal injury risk children with recurrent pneumonia exhibit nearly a 2-fold increase in D-lactate levels, over a 2-fold increase in diamine oxidase levels, and a 20-fold increase in endotoxin levels compared to healthy children, with these significant changes providing quantitative evidence for clinical early warning (92). The quantities of *Bifidobacterium* and *Escherichia coli*, along with the B/E ratio, serve as quantitative indicators of intestinal colonization resistance, assessing the degree of microbiota dysbiosis in children with recurrent pneumonia have a B/E ratio <1, in stark contrast to the B/E ratio >2 in healthy children, with this simple and feasible test providing objective indications for microbiota intervention (92, 93).

Secretory immunoglobulin A and mucin reflect the status of the intestinal immune barrier and chemical barrier, with children experiencing recurrent pneumonia showing a 23% decrease in SIgA levels and a 20% decrease in mucin levels, indicating impaired local intestinal immune function (94). High-throughput microbiota testing based on 16S rRNA sequencing comprehensively reveals microbiota structural characteristics infants with bronchiolitis exhibit significantly reduced intestinal microbiota richness, which correlates with long-term respiratory outcomes, providing a new perspective for prognosis prediction. Monitoring during microbiota intervention is a critical component ensuring precision therapy, with microbiota testing before and after fecal microbiota transplantation objectively evaluating treatment efficacy and guiding therapeutic strategy adjustments. In summary, D-lactate, diamine oxidase, and endotoxin reflect mechanical barrier damage, the B/E ratio reflects colonization resistance, SIgA reflects immune barrier function, and high-throughput sequencing comprehensively analyzes microbiota structure in clinical practice, these indicators should be comprehensively utilized for early identification of intestinal barrier damage and microbiota dysbiosis, guiding precision interventions to ultimately interrupt the vicious cycle of infection-dysbiosis-barrier injury (93). It is important to emphasize that the clinical application of these markers is still exploratory at this stage. Large-scale, prospective, multicenter studies are urgently needed to establish diagnostic thresholds, validate their prognostic value, and demonstrate the incremental benefits of combined testing over conventional clinical assessments before these markers can be recommended for routine use.

## 6 Conclusions and future perspectives

Pediatric pneumonia-diarrhea comorbidity represents a common and complex infectious condition in clinical practice, with its pathogenesis closely linked to gut microbiota dysbiosis and immune disorders mediated by the gut-lung axis. This review systematically summarizes the core mechanisms underlying this comorbidity, respiratory infections remotely induce decreased gut microbiota diversity, reduced beneficial commensal bacteria, and increased colonization by opportunistic pathogens; microbiota dysbiosis leads to abnormal short-chain fatty acid metabolism, subsequently triggering Th1/Th2 immune imbalance, dysregulation of the inflammatory cytokine network, and impairment of mucosal barrier function; inflammatory mediators released following intestinal barrier damage further exacerbate pulmonary inflammation, forming a vicious cycle of infection-dysbiosis-immune disorder-barrier injury.

Clinical laboratory testing plays an irreplaceable role in the diagnosis and management of this comorbidity. Combined detection of inflammatory markers improves diagnostic accuracy and facilitates differentiation of pathogen types, nutritional and immune status indicators help identify high-risk children and guide interventions; intestinal barrier function markers and microbiota testing enable early warning of intestinal injury and monitoring of treatment efficacy.

Looking forward, research in this field could pursue the following directions, employing advanced technologies such as metagenomics to elucidate the functional mechanisms of specific bacterial species in gut-lung axis regulation; conducting large-scale prospective cohort studies to establish causal relationships between microbiota alterations and comorbidity development; developing predictive models based on multi-omics integration for early warning of comorbidity risk; and exploring the clinical translational value of interventions targeting the microbiota-immune axis, including precision probiotics, fecal microbiota transplantation, and short-chain fatty acid supplementation. A deeper understanding of gut-lung axis regulatory mechanisms will pave new pathways for precision prevention and treatment of pediatric pneumonia-diarrhea comorbidity.
